# Supporting Access to Care Through Peripheral Devices and Patient-Generated Health Data: Qualitative Study

**DOI:** 10.2196/88405

**Published:** 2026-04-06

**Authors:** Ashley C Griffin, Caroline Gray, Victoria Ngo, Camila Chaudhary, Stephanie L Shimada, Amanda M Midboe, Donna M Zulman

**Affiliations:** 1 Center for Innovation to Implementation VA Palo Alto Health Care System Menlo Park, CA United States; 2 Center for Biomedical Informatics Research School of Medicine Stanford University Stanford, CA United States; 3 New England Geriatric Research Education and Clinical Center VA Bedford Healthcare System Bedford, MA United States; 4 Center for Health Optimization & Implementation Research VA Bedford Healthcare System Bedford, MA United States; 5 Department of Health Law, Policy, and Management Boston University School of Public Health Boston, MA United States; 6 Department of Population and Quantitative Health Sciences University of Massachusetts Chan Medical School Worcester, MA United States; 7 Department of Public Health Sciences, Division of Health Policy and Management School of Medicine University of California Davis Davis, CA United States; 8 Division of Primary Care and Population Health, Department of Medicine School of Medicine Stanford University Stanford, CA United States

**Keywords:** remote patient monitoring, self-management, health services accessibility, patient generated health data, veterans, mobile phone

## Abstract

**Background:**

In 2016, the US Department of Veterans Affairs (VA) implemented a national initiative to distribute video-enabled tablets and peripheral devices, such as blood pressure monitors and weighing scales, to patients facing geographic, clinical, or socioeconomic challenges. Such patients could potentially benefit from health monitoring in conjunction with video-based care, as peripheral devices offer opportunities to enrich care received during a video visit and support tracking of health-related data collected outside of clinical care, or patient-generated health data. However, little is known about experiences with the devices and how they could support improved access to care.

**Objective:**

We explored patients’ experiences with VA-issued peripheral devices and their impact on video-based care and health monitoring outside of clinical visits.

**Methods:**

We conducted in-depth semistructured interviews among patients who received VA-issued tablets and peripheral devices between 2023 and 2024. Purposive sampling was used to gather views based on gender, age, race or ethnicity, and rurality. Interviews were transcribed and analyzed using rapid qualitative analysis, guided by the Unified Theory of Acceptance and Use of Technology.

**Results:**

Among 25 patients, most received a blood pressure monitor (21/25, 84%), a weight scale (14/25, 56%), and/or a pulse oximetry device (12/25, 48%). The majority reported using their peripheral devices (23/25, 92%) and tablets (19/25, 76%) to monitor their vital signs and attend video visits. Qualitative analysis yielded ten themes reflecting experiences and impacts of the devices, organized by the Unified Theory of Acceptance and Use of Technology constructs: “effort expectancy” consisted of (1) familiar and easy to use devices and (2) challenges of Bluetooth pairing and measurement; “performance expectancy” consisted of (3) integration with video visits, (4) health monitoring for peace of mind, (5) perceptions of improved vital signs and lifestyle behaviors, (6) removing obstacles to in-person care, and (7) desiring an overall picture of health; “social influence” consisted of (8) fostering care team connections and (9) promoting awareness of tablets and peripheral devices; and “facilitating conditions” consisted of (10) supportive help desk infrastructure. Overall, patients described using peripheral devices during virtual visits by syncing data to the tablet for real-time access by their care team. They also reported manually tracking and sharing patient-generated health data with their care team. Despite some challenges with Bluetooth pairing, patients found the devices easy to use and contributed to improved health and motivation. Devices also reduced logistical burdens of in-person visits, especially for those with limited mobility, visual impairments, mental health needs, or transportation barriers.

**Conclusions:**

Patients perceive that peripheral devices can enhance video-based care and support health care access and chronic disease management. Patients reported benefits to health, behavior, and communication with care teams. To maximize the impact, program enhancements should prioritize device interoperability, accessible training, and expanded outreach.

## Introduction

### Background

Multiple forces are aligning to integrate patients’ remote health data into care settings, showing strong potential to improve health outcomes. With 91% of adults in the United States owning a smartphone [1], the proliferation of consumer health technologies presents numerous opportunities for patients to track and monitor their health through apps and peripheral devices, such as blood pressure monitors, weighing scales, or pulse oximetry devices. A growing proportion of the US population is estimated to use a wearable device to monitor their health (40%), of which the majority (73%) are willing to share these data with their care team [2]. In addition to patient interest in tracking and sharing their health data, substantial progress has been made in the remote monitoring regulatory and interoperability ecosystems [3-5]. Notably, the Fast Healthcare Interoperability Resources standard [6] provides a framework to exchange data between health information systems, devices, apps, and electronic health records (EHRs). These regulatory and technological advancements have given rise to a plethora of patient-generated health data (PGHD), or data created and recorded by patients outside of the clinical setting [7].

Efforts within health systems to leverage PGHD are beginning to flourish [8-11], yet numerous studies have identified digital health disparities in access and use of peripheral devices [12-14]. Patients with lower socioeconomic status, limited internet access or connectivity, barriers to care, and older adults have been most impacted by the digital divide. These disparities can impact care and outcomes, as the use of peripheral devices and PGHD has demonstrated early value in personalized medicine and improvements in physiological measures and symptoms across a variety of chronic conditions [9,10,15,16]. Given the value of peripheral devices, system-wide efforts are needed to support greater access and more equitable use to allow all patients to receive these benefits.

To combat digital disparities, the US Department of Veterans Affairs (VA) distributes internet-connected tablets and peripheral devices to VA patients with barriers to accessing in-person care [17]. Patients are eligible for a tablet if they lack a device or connectivity, have a barrier to care (ie, geographical, clinical, or social), and require a VA video-based appointment within the next 90 days. In addition to the tablet, depending on the patient’s clinical needs, they can receive 1 or more peripheral devices including a blood pressure monitor, pulse oximetry device, weighing scale, stethoscope, or thermometer. Peripheral devices are intended to be connected to the tablet via Bluetooth and used during a video visit to share a patient’s vital signs with the care team. The tablets also contain a variety of preinstalled VA apps, allowing patients to engage with the VA patient portal or VA Mobile App Store [18]. Previous work has shown increased registration and use of VA’s patient portal after receiving a tablet [19] and demonstrated how tablets foster opportunities to use VA apps for multiple health management needs [20].

Despite growing evidence supporting remote monitoring and PGHD integration, little is known about patient experiences using peripheral devices in conjunction with video-enabled tablets, and how they use these devices outside of clinical visits to collect and track health data. We also have a limited understanding of how these technologies are experienced by patients who face barriers to care. Previous work has examined patients’ expectations and experiences with remote monitoring across various chronic conditions, highlighting how patients gain knowledge about their condition, feel reassured about their health, and how the devices support self-management and shared decision-making [21]. Patients have also reported reluctance to use new technologies, lack of trust in technology, concerns about costs, and fear of their data being lost or losing face-to-face communication with their care team [21]. Much of the existing telehealth and remote monitoring literature has focused on disease-specific interventions or on patient populations who already have access to technology [22-24].

National health system initiatives designed to mitigate these inequities, such as the VA tablet and peripheral device distribution program, aim to address structural barriers to care by providing devices, connectivity, and technical support to patients with documented challenges [17]. Previous evaluations of this initiative have demonstrated improvements in access to mental health services, continuity of care, and reductions in emergency department visits among VA patients receiving tablets [25,26]. These evaluations have mostly focused on usage and clinical outcomes rather than the patient’s lived experience using peripheral devices and the data generated from them. We extend existing work within the context of patients who experience barriers to accessing health care services, including physical and mental health challenges, socioeconomic stressors, and transportation difficulties. Patients with these challenges potentially stand to gain substantial benefits from peripheral devices.

### Objective

Among VA patients who received a tablet and 1 or more peripheral devices, we aimed to assess their experiences with the devices, including setup, use, technical and social support, and integration with their video-enabled tablet. We also examined how patients used PGHD generated from the peripheral devices to enhance their health, including through health monitoring, communication with care teams, and access to care.

## Methods

### Setting

The VA Office of Rural Health and Office of Connected Care tablet initiative provides tablets (ie, Apple iPads with T-Mobile [Deutsche Telekom] or Verizon [Verizon Communications Inc] data plans) and peripheral devices to VA patients based on an assessment by clinicians and social workers using a Digital Divide Consult template in the VA’s EHR [27]. As of March 2026, more than 10,500 VA patients have been shipped both a peripheral device and a tablet. The primary goal of peripheral device distribution is to use the devices during a video visit, in which devices are paired via Bluetooth to the tablet. During the video visit, data are displayed to the care team within the VA Video Connect app and can be entered into the EHR. Devices can also be used without Bluetooth, but data are not shared with VA care teams when used without Bluetooth. Upon receipt of a device, patients receive a phone call from the VA Connected Device Support Team to assist with setting up and pairing the Bluetooth device with the tablet.

### Ethical Considerations

This evaluation was conducted as part of the Virtual Access Quality Enhancement Research Initiative and was designated as nonresearch by the Stanford University institutional review board (#70128). All patients provided consent to participate in the interview and received a US $50 gift card upon completion. All recruitment and interview data were stored in a private and secure location to ensure confidentiality.

### Sample and Sampling

To identify patients for in-depth semistructured interviews, we used EHR data from the VA Corporate Data Warehouse (CDW) that included sociodemographic and peripheral device characteristics. Patients were eligible if they were shipped a peripheral device between February 2023 and February 2024, and had a physical mailing address, email address, and phone number on file. We used purposive sampling to include perspectives from at least 25% (7/25) of women, older adults (older than 65 y), patients of diverse race or ethnicity, and those who resided in a rural area. Rurality was based on Census tract Rural Urban Commuting Areas codes corresponding to each patient’s ZIP code [28].

We also aimed to gather views among patients who received different devices (ie, blood pressure cuff, weighing scale, pulse oximetry, thermometer, and stethoscope), those who received a device setup call from the VA Connected Device Support Team, and those who received a peripheral device and tablet but did not use it. Setup calls were tracked on a VA dashboard and then linked to our sample in the CDW. Use of the peripheral device and tablet was reported during the interview prescreening process and noted in our recruitment sheet. We also characterized the sample based on the most common chronic physical and mental health conditions from the CDW based on the list from Yoon et al [29].

### Recruitment

Patients were first contacted through an email explaining the project and asking if they were interested in participating. If they expressed interest, they received a phone call to confirm that they had obtained a peripheral device and to schedule an interview. If there was no response to the initial recruitment email, 2 follow-up phone calls were attempted. Recruitment occurred between March 2024 and August 2024.

### Interview Guide Development

To examine patients’ experiences with the devices, an interview guide was developed using constructs from the Unified Theory of Acceptance and Use of Technology (UTAUT) [30], which is a well-established framework to assess acceptance and behavioral intention to use health technologies. Interview questions related to UTAUT constructs were developed to elicit input on various topics, including device setup and use, technical and social support, communication with the care team, integration with the tablet, use of data generated from the device, and improvements in health (Table 1).

**Table 1:** Interview questions mapped to constructs from the Unified Theory of Acceptance and Use of Technology.

Construct	Definition applied to this study	Questions
Effort expectancy	The degree of ease associated with using the peripheral device	What was your experience like setting up the device? What was it like learning to use the device? How often do you use it?
Performance expectancy	The degree to which a patient believes that using the peripheral device will help them to improve their health	Do you consider the device to be useful? (Why or why not?) Have you used the device before or during a video visit or in-person visit with your care team? (If so, please describe how you used the device.) How did receiving a device help you talk with your doctor? How have you used the data generated from the device? Has it resulted in any lifestyle changes? In your opinion, can the device improve your [blood pressure, weight, other health monitoring]?
Social influence	The degree to which a patient perceives that important others believe they should use the peripheral device	Who is supportive of you using the device? Do you have anyone in your support circle (family or friends) who helps you use the device?
Facilitating conditions	The degree to which a patient believes that organizational and technical infrastructure exists to support use of the peripheral devices	Have you had prior training on how to set up and use the device? What did you find most challenging about setting up the device? Using the device? Are you able to get technical support on the device? What other information or support do you need to use the device?

### Interviews

Interviews were conducted by 2 trained qualitative researchers (ACG and CG) using Microsoft Teams software. Each interview included the interviewer and patient, with the exception of 1 interview that also involved the patient’s caregiver and 1 interview with a late patient’s family member. Thus, 26 individuals were interviewed to represent the 25 patients. Demographic information is presented for the 25 patients who received the peripheral devices. Interviews lasted for 30-60 minutes and were audio and/or video-recorded upon consent. Interviews were completed between April 2024 and August 2024.

### Analysis

Interview data were transcribed verbatim by Keystrokes Transcription Service and imported into ATLAS.ti qualitative data analysis software (version 23; Lumivero). Patient narratives within the transcriptions were analyzed using rapid qualitative analysis, guided by the Planning for Assessing Rigor in Rapid Qualitative Analysis framework [31]. One team member (CG) reviewed the transcripts and interviewer’s notes to summarize the content into a matrix based on the initial topics from the interview guide using ATLAS.ti. The matrix was then exported into Microsoft Excel, where a second team member (ACG) reviewed the data summaries and referenced the notes and recordings as needed. The resulting summaries were then discussed between the 2 team members (ACG and CG) and iteratively grouped into themes within Excel using a hybrid inductive-deductive approach to allow for emergent themes. The larger project team also provided input on the themes. Illustrative quotes were identified within each theme. To organize the themes and allow for comparison with related studies, we grouped each theme under the most relevant UTAUT construct.

The study team actively engaged in ongoing reflexive practices throughout data collection and analysis. We are aware that our personal and professional backgrounds shape interactions with patients and the interpretation of findings. Similarly, our role as researchers affiliated with the VA may influence patients’ social desirability by potentially overreporting positive experiences or underreporting challenges with the devices because they perceive the interviewers as part of their care system. The study team had no established relationships with patients, and interviews were conducted using open-ended questions and neutral language. Iterative discussions with our multidisciplinary study team helped us remain aware of our positioning and avoid assumptions when interpreting the data.

## Results

### Patient Characteristics

Among 156 patients contacted, 25 (16%) participated in an interview. The mean age was 62 (SD 11) years, 32% (8/25) were female, 40% (10/25) were Black and/or African American, and 44% (11/25) lived in a rural area. Patients had a range of common chronic physical and mental health conditions (Table 2). Based on the EHR Digital Divide Consult, (1) a mental health diagnosis or participation in therapy (7/25, 28%) or (2) living farther than 30 miles from a VA Medical Center (6/25, 24%) were the most common reasons for receiving a tablet and peripheral device. The majority received a blood pressure monitor (21/25, 84%), weighing scale (14/25, 56%), and/or pulse oximetry device (12/25, 48%) from the VA (Table 2). Nearly all (23/25, 92%) reported using at least one of their peripheral devices. The 2 patients who were unable to use their peripheral devices received only a blood pressure monitor and tablet. They explained that the blood pressure monitor was missing a hardware component (ie, arm cuff and display monitor) that prevented them from taking their blood pressure. Others reported using some of their devices but not all of them due to difficulty with Bluetooth setup, already having their own device at home, or not being sure why they received certain devices. Approximately three-quarters (19/25, 76%) reported using their tablet to do a video visit with their care team. Of the 18 patients who used both the tablet and peripheral devices, 9 (50%) were able to successfully connect them via Bluetooth so that their care team could access data in real-time during a video visit. Of the 6 patients who had not done a video visit, 3 described being unable to operate the tablet despite receiving a phone call from the VA Connected Device Support Team, 2 patients did not recall receiving a support call and were unable to start the video visit on their own, and 1 preferred using their desktop for video visits. According to the VA Connected Device Support Team dashboard, most received assistance to set up the peripheral devices and/or tablet, either by the VA initiating contact (15/25, 60%) or the patient initiating contact (6/25, 24%).

**Table 2:** Sociodemographic, clinical, social, and technological characteristics of patients who received a peripheral device (n=25).

Characteristics					Patients, n (%)
**Sociodemographic**					
	**Age group (y; mean 62, SD 11 y)**				
		18-44	3 (12)		
		45-64	10 (40)		
		65-74	9 (36)		
		75-84	3 (12)		
	**Sex**				
		Female	8 (32)		
		Male	17 (68)		
	**Race or ethnicity**				
		Black and/or African American	10 (40)		
		Hispanic or Latino	2 (8)		
		More than one race	1 (4)		
		Native American or Alaska Native	1 (4)		
		White	10 (40)		
		Unknown	1 (4)		
	**Rurality**				
		Rural	11 (44)		
		Urban	14 (56)		
**Clinical and social**					
	**Physical health conditions**				
		Hypertension	17 (68)		
		Heart failure, heart disease, or atrial fibrillation	9 (36)		
		Hyperlipidemia	16 (64)		
		Diabetes	9 (36)		
		Obesity	8 (32)		
		Lower back pain, fibromyalgia, or chronic migraine	17 (68)		
		Rheumatoid arthritis or osteoarthritis	6 (24)		
		Cataract, glaucoma, or visual impairments	8 (32)		
	**Mental health conditions**				
		Depression	14 (56)		
		Anxiety	13 (52)		
		PTSD^a^	13 (52)		
		Drug or alcohol use disorder	6 (24)		
	**Digital divide consult reasons (clinicians can select multiple reasons)**				
		Mental health diagnosis or participating in therapy	7 (28)		
		Lives father than 30 miles from VA^b^ medical center	6 (24)		
		Health issues make the patient homebound	4 (16)		
		Difficulty attending visits at VA facility (eg, psychological distress or immunocompromised)	4 (16)		
		Work, school, or caregiver commitments make in-person visits challenging	4 (16)		
		Other health issues (ie, hospitalized in last 90 days, hospice diagnosis, requires frequent video visits)	4 (16)		
		Other reasons (ie, social isolation, costs, or difficulty with public transport)	3 (12)		
**Technological**					
	**Peripheral devices (patients can receive multiple devices)**				
		Blood pressure monitor	21 (84)		
		Weighing scale	14 (56)		
		Pulse oximetry device	12 (48)		
		Thermometer	7 (28)		
		Stethoscope	5 (20)		
	Ever used a peripheral device			23 (92)	
	Ever had a video visit on tablet			19 (76)	
	**Tablet and/or peripheral device setup call**				
		VA-initiated setup complete	15 (60)		
		Patient-initiated setup complete	6 (24)		
		Patient requested in-person setup at local VA	2 (8)		
		Declined assistance or did not answer	2 (8)		

^a^PTSD: posttraumatic stress disorder.

^b^VA: US Department of Veterans Affairs.

### Experiences With Peripheral Devices

Overall, patients described using the peripheral devices for a variety of purposes, ranging from monitoring blood pressure post surgery, participating in virtual diabetes management classes, and engaging in home-based primary care programs, among other medical reasons. Patients reported that the devices offered many benefits when used in combination with the tablet, which was the intended use of receiving a peripheral device as part of the VA’s national telehealth initiative. Patients also described valuable aspects of using the devices in between visits and having the ability to track and reflect on their health data over time. They discussed how the devices were simple to set up and use, and they highlighted opportunities to improve their distribution and usability. The following interviews revealed 10 themes reflecting patients’ insights about and impacts of using the peripheral devices (Textbox 1).

**Themes for patient experiences with peripheral devices corresponding to constructs from the Unified Theory of Acceptance and Use of Technology.**

**Effort expectancy**

- Familiar and easy to use devices
- Challenges of Bluetooth pairing and measurement

**Performance expectancy**

- Integration with video visits
- Health monitoring for peace of mind
- Perceptions of improved vital signs and lifestyle behaviors
- Removing obstacles of in-person care
- Desiring an overall picture of health

**Social influence**

- Fostering care team connections
- Promoting awareness of tablets and peripheral devices

**Facilitating conditions**

- Supportive help desk infrastructure

### Effort Expectancy

#### Familiar and Easy to Use Devices

The devices were easy to set up and use, and patients discussed how they had used similar devices before. One patient described,

It was really easy. The first couple of times I learned it and just kept track of everything… I did that [set up] on my own, and the instructions were pretty easy.

P5

Among the 2 caregivers, both reported using similar devices and felt they were easy to operate. The caregiver of a home-based patient explained,

I’ve been taking care of him for a long time because I’m his caregiver plus his wife… I check his blood pressure, his temperature, and write it down in my little tablet. I’m up-to-date on it. You get the hang of everything after a while.

P2

#### Challenges of Bluetooth Pairing and Measurement

The greatest challenge with using the peripheral devices was connecting them to the iPad (Apple Inc) via Bluetooth. One patient felt,

It was just an issue connecting it… And I got frustrated and just used my own scale.

P10

Bluetooth pairing issues were mentioned across all types of peripheral devices, and some patients were unsure if their devices were Bluetooth-enabled. Patients who were unable to pair the device felt that having a Bluetooth connection would be useful so that their care team could see their readings.

There were also some concerns about the accuracy of the devices. Sometimes patients felt their blood pressure readings were too high or too low and brought in their devices to the clinic for help. One person reported,

I think I had been using [the blood pressure cuff] wrong at first because I kept getting these really low readings. So I called them and told them it didn’t work, and when I went in and got to see the nurse in one of my appointments, she showed me how I should be using it. Now that I use it right and more regularly, I feel comfortable about the fact that I’m tracking my blood pressure regularly.

P8

It was recommended to have the option for classes to set up and understand how to take accurate measurements,

Sometimes you need to understand a little more about your blood pressure than just the cuff and the button that you push because it makes a difference on your feet being on the floor and stuff like that.

P24

### Performance Expectancy

#### Integration With Video Visits

Patients described using the peripheral devices during video visits in which the devices were paired via Bluetooth to the tablet. This allowed patients to take their vital signs during the visit and display them automatically on the screen for their care team. These patients were usually part of a VA program, such as home-based primary care or a diabetes management program. One patient explained,

The weight scale is Bluetooth connected to the iPad. So when I step on it, it will just tell me to step off, and it’ll put my weight on the iPad for the provider that I’m talking to so that they get the information. The same thing with the arm cuff… It automatically shows up on the screen.

P4

Patients could also take their vitals right before the visit, with one patient discussing how,

I describe my data to my care team and it automatically sends it to them too. It’s all connected in one system there with the tablet.

P16

The stethoscope and thermometer, while less frequently discussed compared with the other types of devices, were commonly used in real-time during video visits.

#### Health Monitoring for Peace of Mind

Patients found the devices helpful for monitoring and feeling in control of their health. One patient stated,

I feel better and less anxious about my health because I have some control over some of the things. I don’t have to wait for an appointment to get my blood pressure checked or to get weighed in and see if I gained or lost weight. So, it’s just a feeling of more control and it reduces my anxiety and stress.

P8

The devices also provided reassurance for some patients that their medications were working properly and helped them feel less stressed about their blood pressure. Patients mentioned the benefits of monitoring their blood pressure after starting new medications (eg, steroids) and for certain medications that they were instructed not to take when their blood pressure was too low (eg, beta blockers).

The blood pressure monitor, weighing scale, and pulse oximeter were commonly used on a daily basis, such as to check blood pressure before taking medicine, before pacemaker battery replacement, or if something felt off. One patient explained,

On the oximeter, sometimes [I use it] two or three times a day. When I come back in from getting the groceries or whatever, and I have a hard time breathing, I take my oxygen level.

P6

Patients discussed how being able to take their vitals at home gave them comfort in helping to identify or distinguish between different health issues. For example, one patient described using the pulse oximeter to determine the difference between a panic attack versus shortness of breath for physical reasons. They explained how if they were experiencing mild shortness of breath, but their oxygen levels were high, they could use techniques to calm down and then let their doctor know about the episode. One patient also described using the blood pressure cuff to identify a headache from elevated blood pressure versus a migraine,

I get migraines but also get headaches from high blood pressure. So, [the blood pressure monitor] identifies just how high it is, do I need to call someone, and if it’s a headache due to high blood pressure… If my blood pressure is high enough for a headache, I need to go in.

P19

#### Perceptions of Improved Vital Signs and Lifestyle Behaviors

Patients reflected on how the devices made a positive impact on their health and lifestyle behaviors. For example, monitoring blood pressure and weight fostered other healthy habits, such as eating less salt, drinking more water, and walking more. One patient reported,

I have increased my walking and watched what I'm eating, and last month I went to my doctor – he said I had lost 12 pounds.

P17

Others self-reported losing weight or lowering their blood pressure after receiving the devices. Patients also elaborated on how the devices strengthened their motivation to improve their health and helped them stay accountable since a member of their care team was checking on the data. Notably, one person indicated they were able to be taken off their current blood pressure medication, and another described how they did not have to start a medication.

#### Removing Obstacles of In-Person Care

Patients described how taking their vitals at home through the peripheral devices helped them avoid the challenges of attending in-person care. Patients described having mobility and visual impairments, needing to transport medical equipment (eg, oxygen tanks and wheelchairs) or arrange ambulatory transportation. One patient elaborated on using the peripheral devices in combination with video visits, which avoided some of the in-person challenges with their wheelchair.

I’m just very, very thankful that I can go through a video chat rather than have to load my powerchair up every time… it’s kind of rough for me to get all that hooked up, and go up there and unhook and then come out and hook it back up again. And then if it’s raining, I’ve got to fight with the cover that goes over the chair so it doesn’t ruin.

P16

Other patients mentioned how attending in-person visits required them to schedule time off from work or drive long distances, which was especially challenging for some patients with anxiety.

I live in a smaller suburban area and most of my appointments are in the city, so that really gets my anxiety up… Sometimes it’s more beneficial to be able to be in the safety of your own little habitat, to stay and do your appointment there. Sometimes that’s beneficial because you’re not distracted and all worked up and anxious by the time you get there. And then you have to spend half the appointment trying to calm down and focus on what you’re doing there and nervous about having to make the drive back.

P25

#### Desiring an Overall Picture of Health

Patients were interested in viewing combined visualizations of their health within 1 interface, including their weight trends, blood pressure readings, and other vital signs. One patient described,

It’d be nice to be able to see something that I could reflect on over time, even if it was just six months at a time, to see the trends and how it’s going because I suspect that everything’s going to be worse when I’m depressed... Opening an app and putting that information in would be really awesome because then I could look at the whole entire picture.

P19

Patients also reported tracking their peripheral device data in combination with personal device data, such as physical activity, nutrition, or glucose, to reflect on improvements in their health. Another patient stated, “*knowledge is power*” [P24] when referring to their interest and perceived benefit in tracking and visualizing data from devices over time.

### Social Influence

#### Fostering Care Team Connections

Patients explained how using the devices supported communication and connection with their care team. One patient stated,

I believe this [blood pressure monitor] device and having the connection with the physical therapist from cardiology improved my desire to get better. It had an immense effect on not just my mental side but the physical side, as well. And at that time, it had more to do with my mental health more than even the physical health.

P23

Patients also communicated with their care team as a result of data from the devices, such as asking for advice about fluctuating blood pressure measurements or weight. Patients also discussed how a combination of technologies supported communication with their care team. For example, the VA’s My Health*e*Vet patient portal was used to communicate with their care team about data from the devices, such as sending messages or screenshots of their data when they had questions. Others reflected on reviewing their personal health information in the portal and their communication with their care team.

#### Promoting Awareness of VA-Issued Tablets and Peripheral Devices

Patients heard about receiving the tablets and devices from other veterans, which prompted them to reach out to their care team. For example,

I found out about [the devices] through another veteran, and I called right then and got the ball rolling… I try to spread the information as much as I can… The people in my building don’t know that they can get a tablet and make it easier to communicate with the hospital.

P24

Patients recommended that more veterans need to know about this program and that their care team should discuss the opportunity to provide a tablet and any needed peripheral devices to support the veteran.

### Facilitating Conditions

#### Supportive Help Desk Infrastructure

Patients described receiving a phone call from the VA help desk to assist with setting up the peripheral devices to ensure they could take their vitals at home and start a video visit. Patients found the help desk personnel to be very helpful and effective in training them to use the technologies, with one patient stating how,

I feel more confident now that when I go to log on I’m going to get right on [to the video visit].

P8

Patients could also contact the help desk at any time, as a contact number was provided on the back of the peripheral devices. Patients described calling the help desk for support with pairing the device via Bluetooth but were usually not able to set up the Bluetooth connection over the phone. Overall, patients did not need any ongoing technical support with the devices but reported knowing how to reach out to the help desk if any technical issues came up.

## Discussion

### Principal Findings

This qualitative study examined the experiences of patients who received VA-issued peripheral devices and tablets as part of a national telehealth initiative designed to address access barriers to care. Overall, nearly all patients who were interviewed reported using the peripheral devices and discussed the benefits for their health management, improved communication with their care team, and how the devices reduced their burden of attending in-person health care services. Patients frequently used peripheral devices to supplement virtual appointments by syncing data to the tablet for real-time clinician access or by reporting the data to their clinician during the appointment. Despite some usability challenges with Bluetooth data transmission, patients found the devices easy to use and integrated them into their daily routine. These findings support the role of peripheral devices and PGHD in supporting care and reducing access disparities among patients with complex medical and mental health conditions.

Our findings align with previous telehealth and remote monitoring literature, including the enabling role of home-based monitoring, the importance of technical and social support, and ongoing challenges related to interoperability [9,21,22]. Our study, however, extends existing research by demonstrating how and why these dynamics operate within a unique patient population supported by a national digital equity intervention. Unlike previous literature that examines early adopters or patients already engaged in digital health, our cohort consisted of patients identified through an EHR digital divide assessment as having significant barriers to accessing in-person care. These barriers included limited previous access to digital technologies, high prevalence of mental health conditions, mobility limitations, rural residence, and transportation challenges. Although these characteristics have historically been associated with lower engagement in telehealth and patient portals [14,22,32], we found that most patients engaged with the peripheral devices and incorporated them into day-to-day health management.

Guided by UTAUT, we identified several aspects that support the adoption and use of peripheral devices. Patients found the devices were helpful in managing their health (performance expectancy) and described numerous health benefits, including reduced anxiety, increased confidence in their medications, and improved diet and physical activity. These findings resonate with existing work showing that PGHD integration can increase self-management and activation, thereby leading to lifestyle changes and better adherence to treatment plans and medication regimens [33]. Overall, the devices were simple to set up and operate (effort expectancy), which may be attributed to support from the VA’s help desk (facilitating conditions) that was used by 84% (21/25) of patients. However, challenges with Bluetooth pairing and data syncing emerged as key concerns. These technical hurdles of usability and interoperability have been identified in previous studies as limiting factors for the effective adoption of peripheral devices [9,12]. Our findings provide new insight into how PGHD is used in real-world settings where technical integration is incomplete and suggest that PGHD can still retain clinical and relational value despite technical challenges. We also found that care team and social connections shape a patient’s engagement with technology, including relationships with the care team, caregivers who assisted in device use, and fellow veterans who promoted awareness of the program (social influence), in accordance with existing work [34-36].

### Peripheral Devices and PGHD Play a Role in Reducing Barriers to Care

Patients described how peripheral devices and tablets mitigated logistical barriers to in-person care. Patients with physical disabilities, mental health concerns, or long travel distances emphasized how home monitoring tools reduced the burden of attending in-person medical visits. These experiences reinforce the potential of virtual care to reduce care fragmentation and expand access to care for patients who face challenges in receiving in-person care. Patients expressed that avoiding stressful travel to appointments improved their ability to focus during visits. This is particularly important for patients with conditions that may impact driving or navigating public transportation, such as anxiety, limited mobility, or visual impairments. Our qualitative findings build upon our team’s earlier quantitative evaluations of the VA’s tablet distribution program, which showed reduced emergency department visits, improved continuity of mental health care, and demonstrated how tablets are reaching veterans from multiple high-need groups [25,26,37]. They also speak to how remote care and PGHD can foster more equitable access to care [13,38].

Patients also discussed using PGHD to spark conversations with their clinicians. In contrast to previous work documenting patient concerns with peripheral devices leading to less face-to-face time with care teams [21], patients in this study described how using their data helped facilitate more frequent and productive communication with care teams. Patients also noted improved understanding of data presented in the My Health*e*Vet patient portal, which highlights the complementary value of access to different digital tools [19,20]. These findings also touch upon considerations for integrating more technical and health literacy education into remote monitoring programs, as patients expressed interest in more in-depth training on device measurement techniques.

### Opportunities for System Integration

An emergent theme in our study was the desire for an integrated platform that could aggregate and visualize data from different devices over time to facilitate self-management and health insights. Although the key focus of this telehealth initiative was for patients to use the peripheral device during a video visit, patients desired additional ways to use their data in meaningful ways. For example, patients expressed interest in tracking trends across multiple health domains (eg, blood pressure, glucose, and weight), which could enhance their understanding of personal health trajectories. These suggestions reflect a broader push in the informatics community toward integrated personal health records and highlight the importance of designing tools that offer patients a comprehensive view of their health [6,15].

Recently, the VA has prioritized integration of PGHD into research and clinical efforts, with the development of an enterprise-wide PGHD database that incorporates third-party apps, wearables, medical devices, and patient portal data [11]. Taking an interoperable approach, PGHD are standardized using Fast Healthcare Interoperability Resources to ensure data consistency across the different data sources so that it can be effectively linked with VA data. Robust system integration of heterogenous data streams can also set the foundation for future data-driven tools and algorithms that can improve patient care.

### Limitations

These findings should be interpreted within the context of several limitations. First, our sample was purposively selected to include diverse perspectives (eg, age, race, gender, geography, and peripheral device or tablet use) but may not be representative of all VA patients who received peripheral devices. As the majority of patients used their peripheral devices (23/25, 92%) or tablet (19/25, 76%), our findings may overestimate positive experiences, including effort expectancy and ease of use, because nonusers were underrepresented. Study participants, and tablet and peripheral device recipients in general, may also have been more engaged, technologically inclined, or positively disposed toward digital tools than the overall VA patient population. Second, although we recruited patients who received a device within the previous 6 months, perspectives on health improvements may be subject to recall biases, which could impact the accuracy of self-reported improvements. Third, our interview guide focused on the core constructs of UTAUT and did not include topics on privacy, trust, or confidentiality of data generated from the peripheral devices. However, privacy concerns and perceived risks may also influence behavioral intention and use of technology [39,40]. Fourth, we did not conduct member checking to validate patient narratives or themes, but during the interviews, patients were asked to clarify responses as needed to ensure their perspectives were understood and properly documented. The insights generated from this work offer valuable real-world perspectives that can inform the ongoing design, implementation, and expansion of remote monitoring programs and PGHD use across large, national health systems.

### Conclusion

As the use of peripheral devices and PGHD continues to grow, input from patients about their experiences can maximize the benefit of these tools for patients and health systems. Our findings suggest that peripheral devices can enhance health monitoring, reduce care barriers, and foster patient engagement, especially when accompanied by other virtual care tools and responsive technical support. These findings provide evidence to support continued investment in health system–wide connected care initiatives, with emphasis on device interoperability, education on device use, and awareness to ensure equitable access.

## Abbreviations

CDW: Corporate Data Warehouse
EHR: electronic health record
PGHD: patient-generated health data
UTAUT: Unified Theory of Acceptance and Use of Technology
VA: US Department of Veterans Affairs

## Data Availability

The qualitative data generated during this project are not publicly available to protect the privacy of individuals who participated. Deidentified data are available from the corresponding author upon reasonable request, subject to institutional or regulatory approval.
